# Epoxy-Based Copper (Cu) Sintering Pastes for Enhanced Bonding Strength and Preventing Cu Oxidation after Sintering

**DOI:** 10.3390/polym16030398

**Published:** 2024-01-31

**Authors:** Seong-ju Han, Seungyeon Lee, Keon-Soo Jang

**Affiliations:** Department of Polymer Engineering, School of Chemical and Materials Engineering, The University of Suwon, Hwaseong 18323, Gyeonggi-do, Republic of Korea

**Keywords:** Cu sintering, epoxy, acidic additive, electrical conductivity, shear strength

## Abstract

The investigation of interconnection technologies is crucial for advancing semiconductor packaging technology. This study delved into the various methods of achieving electrical interconnections, focusing on the sintering process and composition of the epoxy. Although silver (Ag) has traditionally been utilized in the sintering process, its high cost often precludes widespread commercial applications. Copper (Cu) is a promising alternative that offers advantages, such as cost-effectiveness and high thermal and electrical conductivities. However, the mechanical robustness of the oxide layers formed on Cu surfaces results in several challenges. This research addresses these challenges by integrating epoxy, which has advantages such as adhesive capabilities, chemical resistance, and robust mechanical properties. The chemical reactivity of the epoxy was harnessed to both fortify adhesion and inhibit oxide layer formation. However, the optimal sintering performance required considering both the composite composition (20 wt% epoxy) and the specific sintering conditions (pre-heating at 200 °C and sintering at 250 °C). The experimental findings reveal a balance in the incorporation of epoxy (20 wt%) for the desired electrical and mechanical properties. In particular, the bisphenol A epoxy (Da)-containing sintered Cu chip exhibited the highest lab shear strength (35.9 MPa), whereas the sintered Cu chip without epoxy represented the lowest lab shear strength of 2.7 MPa. Additionally, the introduction of epoxy effectively curtailed the onset of oxidation in the sintered Cu chips, further enhancing their durability. For instance, 30 days after sintering, the percentage of oxygen atoms in the Da-containing sintered Cu chip (4.5%) was significantly lower than that in the sintered Cu chip without epoxy (37.6%), emphasizing the role of epoxy in improving Cu oxidation resistance. Similarly, the samples sintered with bisphenol-based epoxy binders exhibited the highest electrical and thermal conductivities after 1 month. This study provides insights into interactions between epoxy, carboxylic acid, solvents, and Cu during sintering and offers a foundation for refining the sintering conditions.

## 1. Introduction

The intensifying demand for high-performance electronic devices necessitates continual advancements in semiconductor packaging technology, which is a critical component in semiconductor manufacturing and design [1]. As a pivotal element, semiconductor packaging acts as a protective container that houses discrete semiconductor devices, dies, or integrated circuits (ICs) and significantly influences power, performance, and the cost of the overall electronic system [2,3].

Semiconductor packaging serves to secure the delicate semiconductor device and to enable electrical interconnections [4,5]. These interconnections are important, facilitating communication and interaction between the semiconductor device, such as IC, and the external circuitry, such as a printed circuit board (PCB) [6,7]. These electrical interconnections can be achieved by several methods, such as wire bonding, flip chip bonding, tape automated bonding (TAB), through-silicon via (TSV), solder ball connection, and sintering [8,9,10,11,12,13]. Each method harbors its unique set of advantages and disadvantages, and their selection is contingent on various factors including their application, performance requirements, cost, and reliability.

(1) Wire bonding is a prevalent method employing thin wires (Au or Al) attached using ultrasonic, thermosonic, and thermocompression techniques. While cost-effective, versatile, and well-established [14], it faces limitations in connection density and signal speed, impacting its integration into compact devices. (2) Flip chip bonding involves inverting the semiconductor die and directly attaching it to the substrate or PCB using solder bumps, allowing for a higher number of connections and improved electrical performance compared to wire bonding [15]. (3) TAB utilizes a tape with conductive traces for connecting the bond pads on the IC to the external lead frame [11]. This is achieved through inner lead bonding followed by outer lead bonding after the device is encapsulated. (4) TSVs are used in 3D IC packaging, where vertical electrical connections are formed through a silicon wafer or die [16]. This allows for the stacking of multiple dies and enhanced electrical performance. (5) Solder ball connection, particularly in ball array packages, ensures a high connection density and reliability between the semiconductor device and the PCB [17]. (6) Sintering involves the use of metal particles (such as Ag or Cu) subjected to heat and pressure. Sintering forms interconnections by bonding particles, thereby creating an electrically conductive path [18].

**Figure 1:**
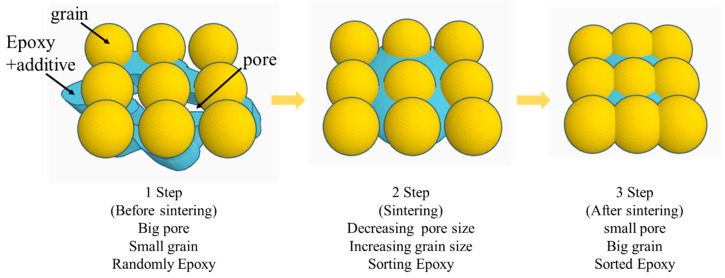
Sintering process with epoxy-based sintering paste.

Among these methods, sintering is often used in applications requiring high thermal conductivity, lead-free environments, and high-temperature stability [18]. The sintering process leads to bonding by connecting particles under a high temperature and pressure without melting them to the point of liquefaction [19], as shown in Figure 1. Ag was initially used for sintering owing to its precious noble metal properties [20,21]; however, its high cost posed a barrier to commercialization. In contrast, Cu, being cost-effective and possessing high electrical and thermal conductivity, is an appealing alternative [22,23]. However, Cu is a non-noble metal and produces oxide layers on its surfaces. The oxide layers should be removed by the combination of reducing additives and solvents, prior to bonding [24]. Cu sintering requires an elevated temperature and pressure, such as >10 min at 300 °C or >60 min at 250 °C, to achieve high electrical and mechanical properties [25]. In addition, after sintering, oxide layers can form, owing to heat, oxygen, and moisture in the air.

Epoxies are extensively used in various applications, ranging from adhesives to coatings, owing to high chemical reactivity, versatile adhesion to diverse substrates, good chemical resistance, and robust mechanical properties [26]. Despite the poor leaving group of -OR on the oxirane ring, the restricted angle of ca. 60°, derived from 109.5° which is the most sterically stable, is subjected to high reactivity, producing hydroxyl moieties. The generated hydroxyl groups exhibit excellent adhesion to various substrates, including Cu [27,28]. Epoxy is routinely cured with hardeners, such as amines, anhydrides, and diacids [29]. Acidic moieties assist in the removal and prevention of the metal oxide layer.

In this study, an intricate interplay between epoxies and acidic additives was examined. The resulting chemical reactions foster strong adhesion and inhibit the formation of oxide layers. However, the sintering performance and electrical properties can be compromised by organic residues after sintering and crosslinked organic components before and during sintering [30,31,32]. Therefore, not only the combined composition but also the sintering conditions are imperative. Different structures of epoxy and solvents under different sintering conditions are employed to unravel the complexities of this interaction.

## 2. Experimental

### 2.1. Materials

**Figure 2:**
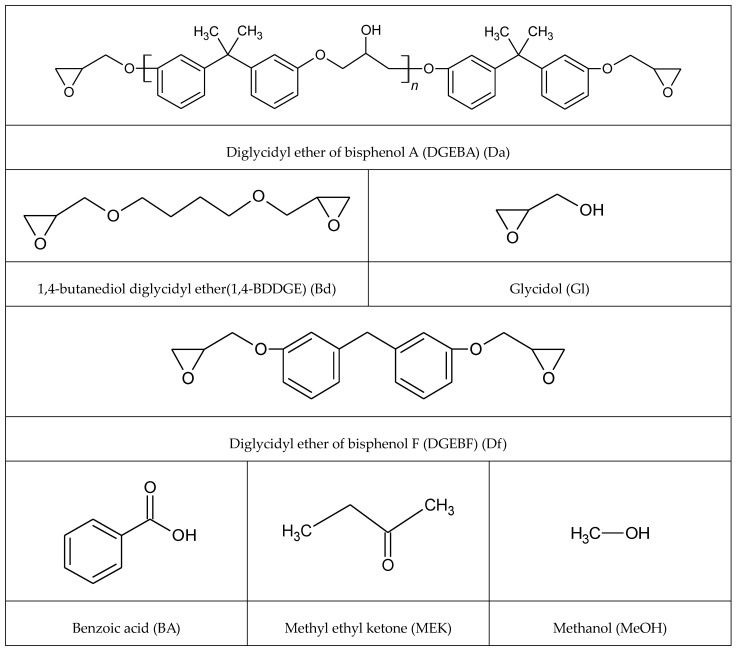
Structural formula of epoxy, solvent, and acidic additives for fabrication of Cu sintering paste.

Cu powder (0809DX, Sky Spring Nanomaterials, Inc., Houston, TX, USA) with a diameter of 1 µm and purity of 99% was utilized to fabricate Cu sintering paste. Epoxy compounds, utilized as additives for enhancing adhesive strength, included diglycidyl ether of bisphenol A (DGEBA (Da), YD-128, EEW = ca. 187 g/eq, Kukdo Chem Co., Seoul, Republic of Korea); 1,4-butanediol diglycidyl ether (1,4-BDDGE (Bd), Kukdo Chem Co., Seoul, Republic of Korea); glycidol ((Gl), Sigma-Aldrich, Burlington, MA, USA); and diglycidyl ether of bisphenol F (DGEBF (Df), YDF-170, EEW = ca. 170 g/eq, Kukdo Chem Co., Seoul, Republic of Korea). These epoxies were chosen to study variations in sintering paste fabrication due to differences in molecular weight, number of epoxide groups, presence of aromatic rings, and type of bisphenol (A vs. F). Additionally, benzoic acid (BA, Tokyo Chemical Industry Co., Tokyo, Japan) was utilized as an activator to eliminate Cu oxide layers, preventing crosslinking with epoxide moieties, owing to monofunctional groups. The Cu oxide layers hindered sintering, owing to their higher melting point of 1330 °C (Cu: 1083 °C). 2-butanone (methyl ethyl ketone; MEK, Samchun Pure Chemical Co., Gyeonggi-do, Republic of Korea) and methanol (MeOH, BNO Chem Co., Cheongju, Republic of Korea) were used as a solvent to conduct comparisons in terms of vapor pressure. All chemicals used in this study are displayed in Figure 2.

### 2.2. Fabrication of Cu Sintering Pastes

Epoxy and acidic additives were mixed in the following ratios, with the solvent being mixed to its maximum saturation point: 0:100, 20:80, 40:60, 60:40, and 80:20 wt%, as shown in Table 1. This mixture underwent a 30 min mixing using a vortex mixer to ensure homogeneity. A total of 80 wt% of Cu powder was then combined with the additives (20 wt %: epoxy + acidic additive and 450 mg/mL of solvents) and subjected to one more 30 min mixing.

### 2.3. Fabrication of Sintered Cu Pastes

**Figure 3:**
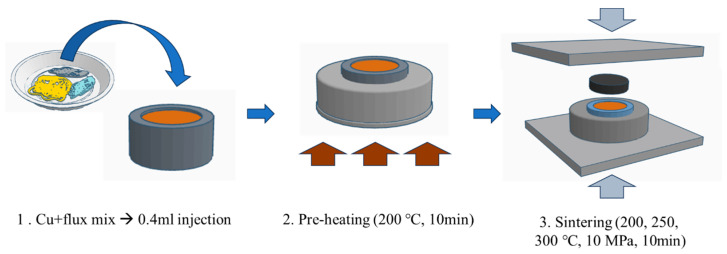
Manufacturing of the sintering paste (reducing solution with Cu) and sintering. The brown color indicates the Cu sintering paste.

The fabricated sintering paste (0.4 mL) comprising the acidic additive, solvent, and Cu was injected into a jig, which was pre-heated without compression at 200 °C (internal temperature: ca. 150 °C) for 10 min, as shown in Figure 3. This pre-treatment allowed for the volatilization of the solvent, the additives that reacted with the Cu, and the unreacted additives, preventing defects that reduce conductivity and mechanical strength. Subsequently, the jig was sealed and sintered under a pressure of 10 MPa for 10 min at various temperatures (200, 250, or 300 °C). The sintered sample in the jig was then cooled to room temperature (22–24 °C) and opened to retrieve the sintered chips.

### 2.4. Characterization

#### 2.4.1. Thermal Properties

Differential scanning calorimetry (DSC; DSC25, TA Instruments Inc., New Castle, DE, USA) was utilized to analyze the thermal characteristics of the sintering pastes as a function of epoxy content. Pre-heating and sintering temperatures were determined using DSC25 [33]. A total of 3 mg of the sample was hermetically sealed in a DSC pan under an ambient environment. It was scanned from −50 to 370 °C at a heating rate of 10 °C/min under nitrogen purging. The DSC analysis was performed using the DSC software (TA Instruments Trios v5.1.1.)

A thermogravimetric analysis (TGA; Perkins Elmer Co., Waltham, MA, USA) was performed for determining the pre-heating and sintering temperatures. Approximately 1.5 mg of the sample was heated from 50 to 500 °C at a heating rate of 10 °C/min under nitrogen purging.

#### 2.4.2. Electrical and Thermal Conductivities

A four-point probe (MCP-T370, Nittoseiko Analytech Co., Kanagawa, Japan) was employed to assess the electrical characteristics of the Cu chips after sintering. The surface resistance was measured, and the correction factor (C) was determined to be 3.98 (D/S = 8; D = 12 mm, S = 1.5 mm), as listed in Table 2 [34]. The electrical conductivity was determined, based on the obtained values.

A thermal conductivity analyzer (TPHS-1, Yeonjin S-Tech Co., Seoul, Republic of Korea) was utilized to assess the thermal conductivity of the sintered chips. The analyzer was calibrated using a flat aluminum chip with dimensions of 50 mm × 50 mm × 10 mm and a thermal conductivity of 170 W/m·K prior to the measurement. Flat cylindrical specimens with a diameter of 40 mm and thickness of 3 mm were used. Two specimens were attached to the prober at 22–24 °C and measured for 40 s under the resistance of 5.2 Ω and power of 0.2 W. The samples were heated to approximately 28 °C due to the generated heat during measurement.

#### 2.4.3. Morphology

Scanning electron microscopy (SEM, Apreo, FEI Co., Hillsboro, OR, USA) was used to evaluate the surface and internal structures of the sintered chips. The degree of sintering, residual organics, and pore sizes were investigated. SEM was performed at a voltage of 10 kV and a current of 6.4 A without Au coating.

#### 2.4.4. Atomic Analysis

**Figure 4:**
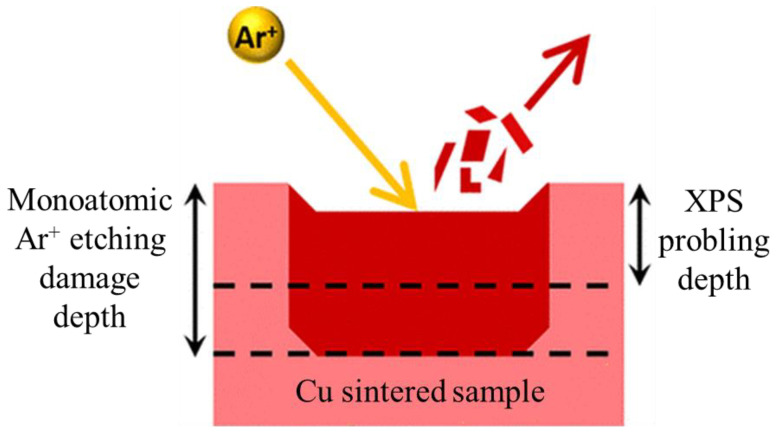
XPS depth profiling etching for composition analysis [38].

X-ray photoelectron spectroscopy (XPS, K-Alpha Plus, Thermo Fisher Scientific Co., Waltham, MA, USA) was employed to conduct depth profiling, and the composition of each layer was compared to confirm the presence of organic residues [36,37]. As shown in Figure 4, the surface of the copper sintered chip was etched in the area, with dimensions of 0.5 cm horizontally × 0.2 cm vertically, using a 400 µm-sized ion beam, and 10 layers were etched, and each etching time was 60 s. The peaks at 285.0, 531.0, and 932.6 eV in the 10th layer represent the binding energies of Cu 2p, C 1s, and O 1s, respectively, and the composition was analyzed through these binding energies. The atomic % measured was used to calculate Cu/O, which enabled the verification of the degree of oxide layer removal.

X-ray diffraction (XRD, ARL Equinox 3000, Thermo Fisher Scientific Inc., Waltham, MA, USA) was utilized to verify the degree of oxide layer removal by detecting the bonding of Cu_2_O. This was measured at 40 kV of voltage and 30 mA of current under an argon gas, and more accurate measurements were obtained by tilting at approximately 30°.

#### 2.4.5. Mechanical Properties

**Figure 5:**
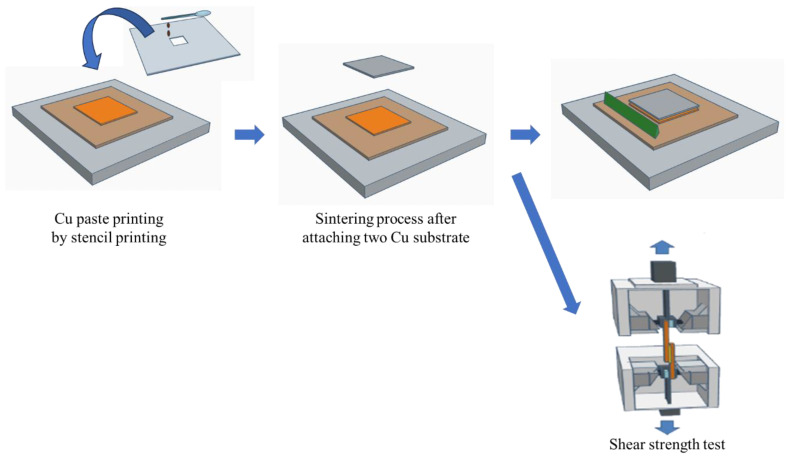
Cu paste stencil printing, sintering, and shear bonding strength test using UTM (DUT-500CM).

A universal testing machine (UTM, DUT-500CM, Daekyung Engineering Co., Seoul, South Korea) was utilized to conduct a lab shear strength test of the Cu sintering paste between two Cu substrates at an elongation speed of 5 mm/min. A Cu substrate with the dimensions of a width of 20 mm, height of 40 mm, and thickness of 20 mm was used, as shown in Figure 5. The Cu sintering paste was stencil printed on the lower Cu plate with the dimensions of 20 × 20 mm^2^ using a stencil-printing substrate on one side. Subsequently, pre-heating was conducted for 3 min. A Cu top plate was covered, and the main sintering adhesion process was conducted for 5 min under a pressure of 10 MPa at 250 °C [39]. The sample was cooled to room temperature and kept for 30 min, followed by the lap shear strength test. The UTM analysis was performed using the UTM software (Testone T0-100s v12.7.2.)

## 3. Results and Discussion

### 3.1. Miscibility and Stability of Cu Pastes

**Figure 6:**
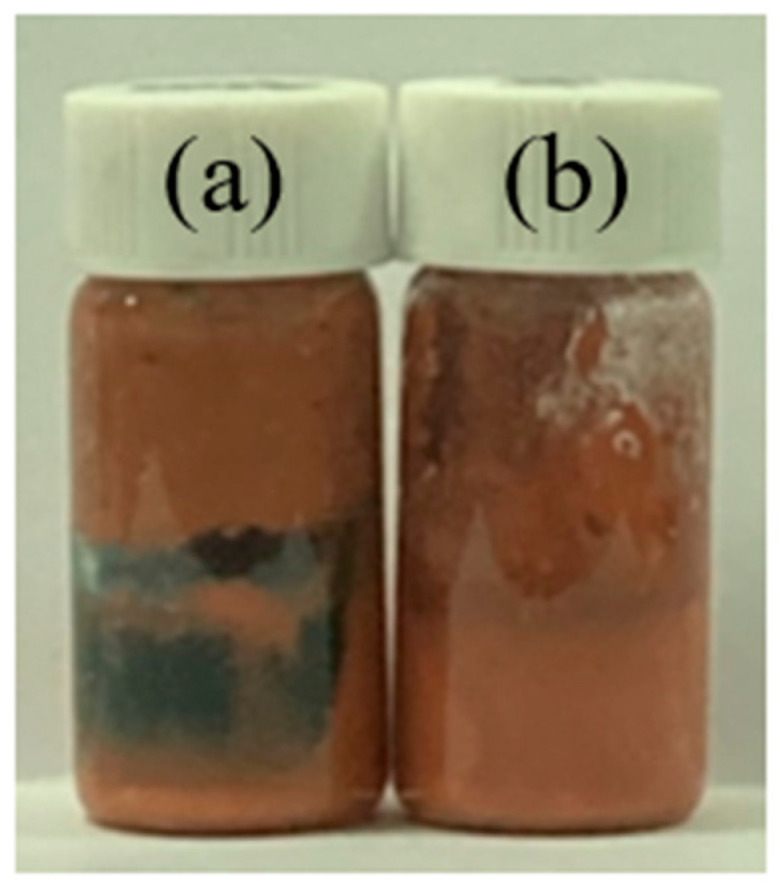
Cu sintering paste: (**a**) Method 1—Cu powder and additives were mixed first and then the solvent was added; (**b**) Method 2—solvent, acidic additive, and epoxy were mixed first, and then the Cu was added.

The Cu powder, epoxy, acidic additive, and solvent were mixed into various formulations to examine the stability of the Cu paste. The mixing procedures were studied through two methods: In Method 1, the Cu and additives were mixed before adding the solvent, while in Method 2, all organic additives and the solvent were mixed prior to adding Cu. Figure 6 shows that Method 1 resulted in a greenish tint and phase separation in the Cu paste [40]. On the other hand, Method 2 exhibited higher mixture stability, devoid of any phase separation.

**Figure 7:**
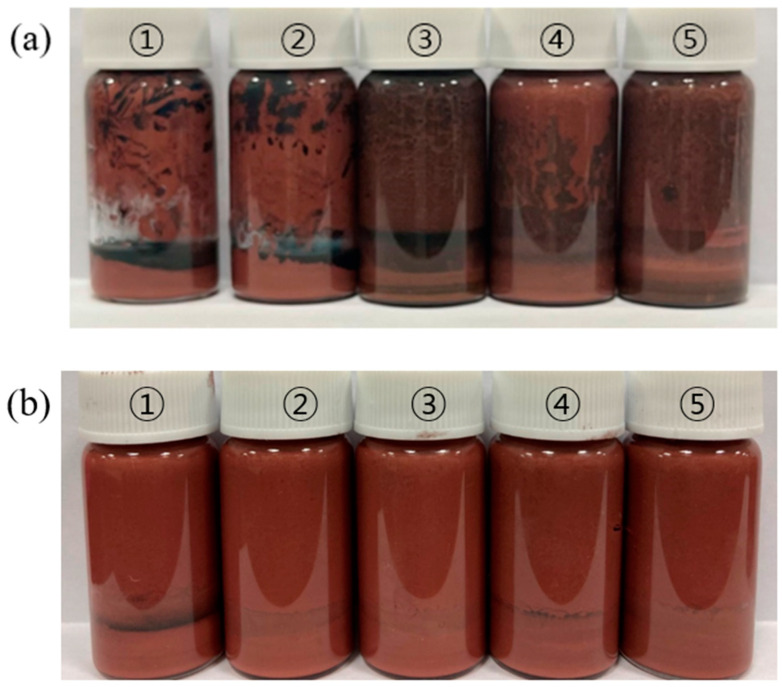
Phase separation of Cu sintering pastes after 24 h with different solvent concentrations: (**a**) 225 mg/mL (acidic additive/solvent) and (**b**) 450 mg/mL (acidic additive/solvent). ① Without epoxy; ② DGEBA (Da); ③ 1,4-BDDGE (Bd); ④ glycidol (Gl); and ⑤ DGEBF (Df).

Upon leaving the Cu sintering pastes with different solvent concentrations at room temperature for 24 h, phase separation was observed. Figure 7a,b depict the Cu sintering pastes with acidic additive/solvent ratios of 225 and 450 mg/mL, respectively. The Cu sintering pastes comprising 225 mg/mL exhibited phase separation due to continuous redox reactions with oxygen. On the other hand, doubling the acidic additive concentration removed the oxide layers, preventing oxidation and ensuring a stable mixture, as is evident in Figure 7b. Thus, a concentration of 450 mg/mL was chosen for further investigation.

**Figure 8:**
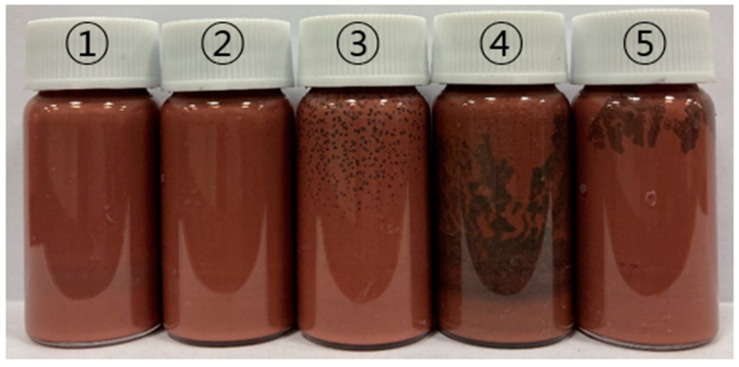
Phase separation of Cu sintering paste containing 450 mg/mL (acidic acid/solvent) with different epoxy/acidic additive ratios: ① 0:10, ② 2:8, ③ 4:6, ④ 6:4, and ⑤ 8:2.

The Cu sintering paste containing 450 mg/mL (acidic additive/solvent) was fabricated with different epoxy/acidic additive ratios (0:10, 2:8, 4:6, 6:4, and 8:2), as shown in Figure 8. Phase separation was compared 24 h after mixing. All the samples exhibited minimal phase separation.

**Figure 9:**
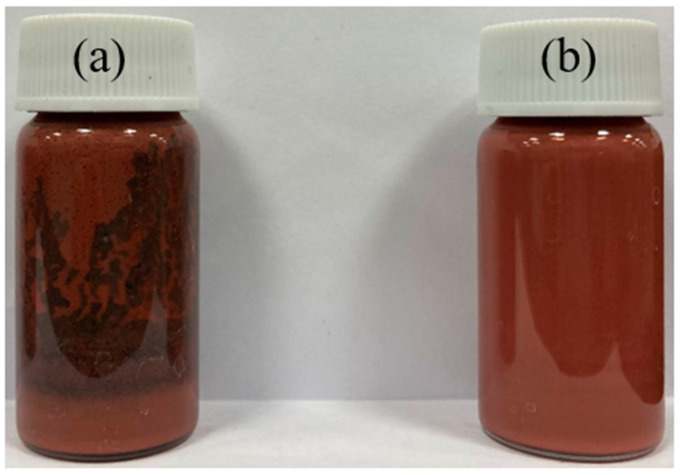
Phase separation of Cu sintering paste seven days after fabrication depending on storage conditions: (**a**) room temperature and (**b**) 4 °C.

The Cu sintering paste mixed at a ratio of 450 mg/mL (acidic additive/solvent) and a 2:8 ratio between the epoxy and acidic additive was stored for seven days at room temperature and 4 °C, as shown in Figure 9a and 9b, respectively. Phase separation was observed in the Cu sintering paste stored at room temperature, whereas the relatively stable Cu sintering paste stored at 4 °C was achieved without phase separation. The oxidation-reduction reaction of Cu caused the green phase separation.

### 3.2. Thermal Properties of Cu Pastes

The thermal properties of the Cu sintering paste were analyzed to determine the optimal pre-heating and sintering conditions. Before sintering, it is essential to ensure maximum evaporation of the acidic additive and solvent during the pre-heating to prevent electrical connection defects during sintering. Moreover, a slight curing of epoxy with a mono-acid (benzoic acid) is required, as complete curing can lead to imperfections.

**Figure 10:**
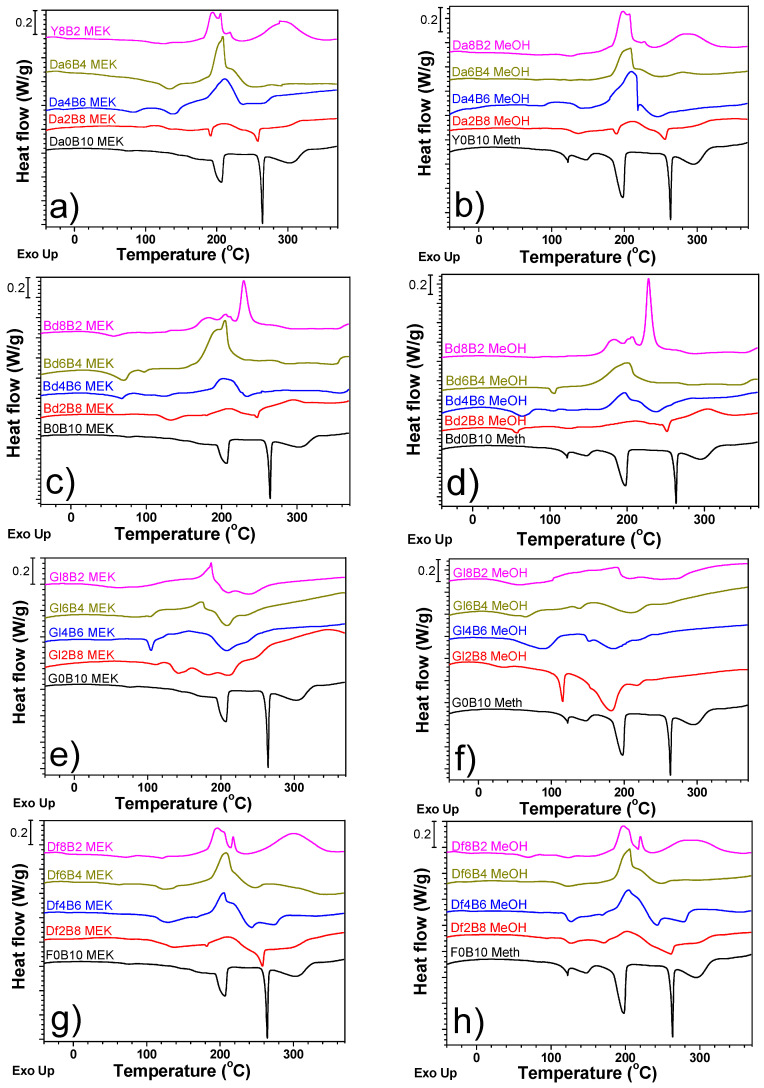
DSC curves of Cu sintering pastes with different solvents, epoxy binders, and epoxy/acid ratios. (**a**,**c**,**e**,**g**): MEK; (**b**,**d**,**f**,**h**): MeOH. (**a**,**b**): Da; (**c**,**d**): Bd; (**e**,**f**): Gl; and (**g**,**h**): Df.

The thermal properties of the epoxy/benzoic acid (BA)/solvent systems with various ratios were analyzed using DSC, as shown in Figure 10. The exothermic peaks of Da8B2 around 200 and 300 °C are indicative of the epoxy-BA reaction and epoxy homopolymerization, respectively. The exothermic enthalpy gradually decreased with a decreasing epoxy/BA ratio. Beyond Da8B2, the exothermic peak associated with homopolymerization vanished, because all epoxy binders (Da) were consumed by the reaction with BA. The effect of solvent type was rarely observed. In the regime beyond Da4B6, endothermic peaks around 200 and 250 °C, associated with the melting and boiling point of BA, respectively, were observed. The intensities of the associated peaks increased with an increasing BA loading. At lower BA concentrations, both the melting and boiling points were elusive, stemming from the full dissolution of BA in the epoxy/solvent mixture and its subsequent reaction with the epoxy, leading to the exhaustive consumption of acidic components. The endothermic peaks contributing to the boiling point of the solvents were not sufficiently sharp and small to be determined, because the DSC pan is usually not completely sealed, even when a hermetical DSC pan is used. The solvent was mixed with other components, and thus, the amount of solvent was low, and the interactions among the organic chemicals influenced the DSC scanning results. Other Cu sintering pastes containing different diglycidyl-based epoxy binders mirrored the results observed in the Da/BA-embedded Cu sintering paste. In contrast, the Cu sintering paste containing a mono-glycidyl-based epoxy binder, specifically Gl, exhibited different behaviors. The oxirane groups produce secondary hydroxyl moieties by reacting with carboxylic acids. Given that Gl comprises both one hydroxyl and one epoxide group, it transitions to two hydroxyl groups, thereby forming a diol. Such a structural transformation potentially affects the DSC curve, which is corroborated by the intricate peaks shown in Figure 10e,f. The Cu sintering paste containing MeOH exhibited more hydrogen bonding and dipole–dipole interactions among the chemicals in the paste, thereby exhibiting strong and neat peaks, as shown in Figure 10f.

**Figure 11:**
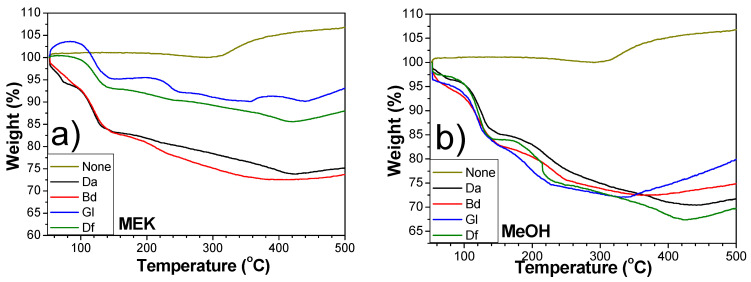
TGA curves of Cu sintering pastes with epoxy/acid (2:8): (**a**) MEK and (**b**) MeOH.

In addition to the evaporation of organic chemicals, the oxidation–reduction dynamics of Cu are crucial. Cu is subjected to oxidation in air. The resultant Cu oxide layer should be removed before and during sintering. Cu oxidation should also be prevented during and after sintering. TGA was utilized to scrutinize the curing and thermal behaviors and to ascertain the optimal pre-heating and sintering parameters. Figure 11a,b depict the Cu sintering pastes containing an epoxy/BA ratio of 2:8, using MEK and MeOH, respectively, as representatives. The initial weight reduction was attributed to the evaporation of the solvent, followed by a more gradual weight loss due to the decomposition of organic compounds. Upon reaching temperatures exceeding 400 °C, a weight gain was observed, which was linked to the formation of Cu oxidation layers by heat. For the Cu sintering pastes formulated with Da and Df, the weight increased at comparatively elevated temperatures relative to the pastes containing alternative epoxy types, indicating high thermal stability. The hydroxyl moieties formed from the reaction of epoxide groups can prevent Cu oxidation at raised temperatures.

Based on the DSC and TGA results, the pre-heating temperature was determined between the melting point of BA (150 °C) and the exothermic peak (200 °C), which is ascribed to curing. During this pre-heating stage, it is essential to ensure the complete evaporation of the solvent while only partially removing the acidic additive. This pre-heating process was performed using a jig positioned on a hot plate, which was regulated by a temperature sensor/controller. Notably, the in-jig sample temperature generally runs 40–50 °C cooler than the set temperature of the hot plate. Therefore, even when the temperature of the plate was set to 200 °C, the actual sample temperature hovered at around 150–160 °C. The sintering temperature was capped below 400 °C before the formation of the Cu oxide layers by heat [41]. Therefore, various sintering temperatures were compared at 200, 250, and 300 °C to determine the optimal sintering conditions with the best sintering outcomes. The Cu sintering paste comprised the Cu powder, mono-functionalized acid, bi-functionalized epoxy binder, and solvent. During the sintering process, the Cu powders bonded together, whereas the acid and solvent evaporated. After sintering, the resultant Cu chip primarily consisted of a network of interconnected Cu paths stabilized by the epoxy. It is important to note that a portion of the acid served to eliminate and inhibit oxidation, another fraction reacted with the epoxy binders, and any remaining acid was expected to evaporate.

### 3.3. Electrical and Thermal Conductivities of Sintered Cu Chips

**Figure 12:**
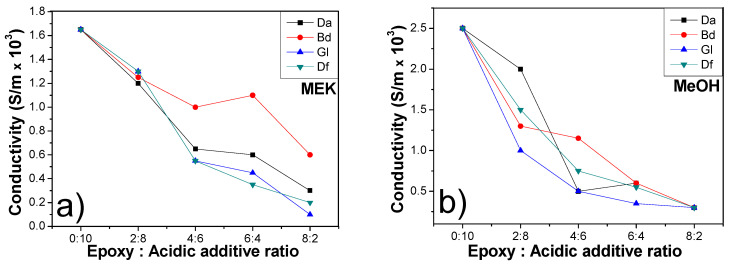
Electrical conductivities of sintered (pre-heating: 200 °C for 10 min, sintering: 250 °C under 10 MPa for 10 min) Cu chips with different epoxy/BA ratios and different epoxy binders: (**a**) MEK and (**b**) MeOH.

The key performance indicators of sintered Cu chips are their electrical and mechanical properties. While the incorporation of epoxy can potentially enhance the mechanical robustness and bonding characteristics, it may compromise the electrical conductivity after sintering. The residual epoxy-based components can impede the electrical pathways. Thus, an optimal balance between the electrical and mechanical properties is crucial when determining the right ratio. As illustrated in Figure 12, the electrical conductivity of the sintered Cu chips gradually decreased as a function of the epoxy ratio of the Cu sintering paste, irrespective of the solvent type. For example, in the epoxy/BA compositions augmented with MEK, a variation in electrical conductivity was observed, corresponding to different ratios. Specifically, these were measured as 1.65 × 10³ S/m for a 0:10 ratio, 1.20 × 10³ S/m for 2:8, 0.65 × 10³ S/m for 4:6, 0.60 × 10³ S/m for 6:4, and 0.30 × 10³ S/m for an 8:2 ratio mixture. In particular, the electrical conductivities of the epoxy/BA ratio of 4:6 were roughly half of those devoid of an epoxy binder. Based on these observations, an epoxy/BA ratio of 2:8 was standardized across all the samples for subsequent evaluations.

**Figure 13:**
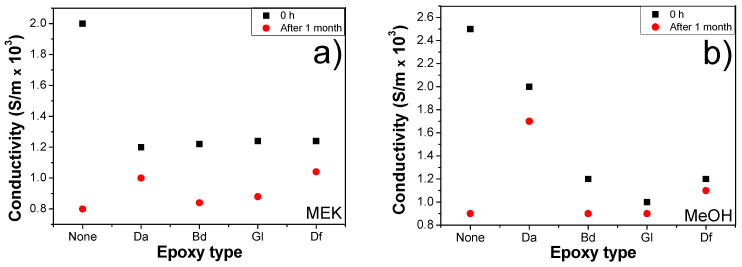
Electrical conductivities of sintered (pre-heating: 200 °C for 10 min, sintering: 250 °C under 10 MPa for 10 min) Cu chips with different epoxy binders at a 2:8 ratio of epoxy/BA immediately and 1 month after sintering: (**a**) MEK and (**b**) MeOH.

We investigated the electrical conductivities of sintered copper (Cu) chips incorporated with various epoxy binders at a ratio of 2:8 (epoxy to BA) immediately and 1 month after production. This aimed to evaluate the oxidation prevention capabilities over time following sintering, as illustrated in Figure 13. The conductivity of the sintered Cu chips devoid of epoxy markedly decreased after 1 month, whereas those containing epoxy binders exhibited only a minor reduction. Especially, the conductivity of the samples using Da and MeOH after 1 month remained substantially higher compared to those without any epoxy. In the samples devoid of the epoxy, the electrical conductivity of the chip immediately after fabrication was 2.5 × 10³ S/m and decreased to 0.9 × 10³ S/m after a duration of 1 month. In contrast, the samples incorporating the epoxy exhibited an initial electrical conductivity of 2.0 × 10³ S/m, which moderately reduced to 1.7 × 10³ S/m over the same period. The lowest rate of conductivity loss was recorded in the samples incorporated with Da or Df, owing to their relatively higher thermal stability. The epoxy resins (epoxy binder + acidic activator) underwent partial curing, which prevents Cu oxidation under ambient conditions after sintering.

**Figure 14:**
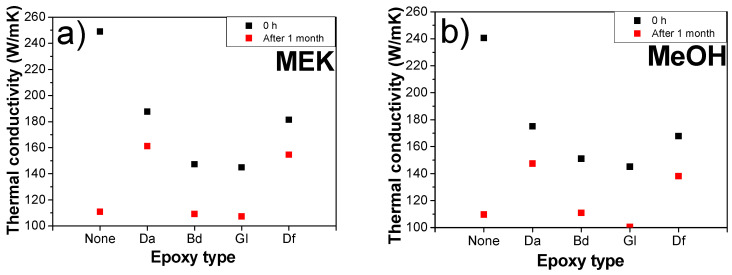
Thermal conductivities of sintered (pre-heating: 200 °C for 10 min, sintering: 250 °C under 10 MPa for 10 min) Cu chips with different epoxy binders at a 2:8 ratio of epoxy/BA immediately and 1 month after sintering: (**a**) MEK and (**b**) MeOH.

The thermal conductivity in the field of sintering is important because of several factors, such as heat distribution, energy efficiency, thermal stress management, and the processes of densification and grain growth. An uneven or insufficient heat distribution results in heterogeneous sintering, ultimately causing defects. High thermal conductivity facilitates reaching the required sintering temperature with low energy, thereby leading to reduced energy costs and efficient sintering. Densification and grain growth can be influenced by thermal conductivity. Differential thermal expansion caused by low and varying thermal conductivities leads to thermal stress during sintering, thereby resulting in cracking and warping. The thermal conductivities of the samples under the identical compositions and conditions as those used in electrical conductivity tests were analyzed, as shown in Figure 14. The immediately sintered sample without an epoxy binder initially represented the highest thermal conductivity among all the samples. However, its thermal conductivities dramatically decreased after 1 month. For instance, the sample fabricated with MEK, devoid of epoxy, immediately after fabrication, exhibited a thermal conductivity of 249.0 W/m∙K, which then declined to 110.9 W/m∙K after a period of one month. Additionally, the sample prepared with the epoxy (Da) demonstrated an initial thermal conductivity of 187.6 W/m∙K, decreasing to 161.2 W/m∙K over the same timeframe. This is because of the absence of an epoxy binder, whereas those containing epoxy demonstrated only a slight decrease in thermal conductivity after the same duration. Especially, the Da- and Df-embedded sintered samples exhibited the highest thermal conductivities after 1 month due to the presence of the bisphenol-type epoxy binders. This preservation of thermal conductivity is attributed to the presence of organic residues formed by reactions between epoxide and acidic moieties. These residues play a pivotal role in preventing oxidation during storage and use, thereby maintaining the thermal properties over time.

### 3.4. Morphology of Sintered Cu Chips

**Figure 15:**
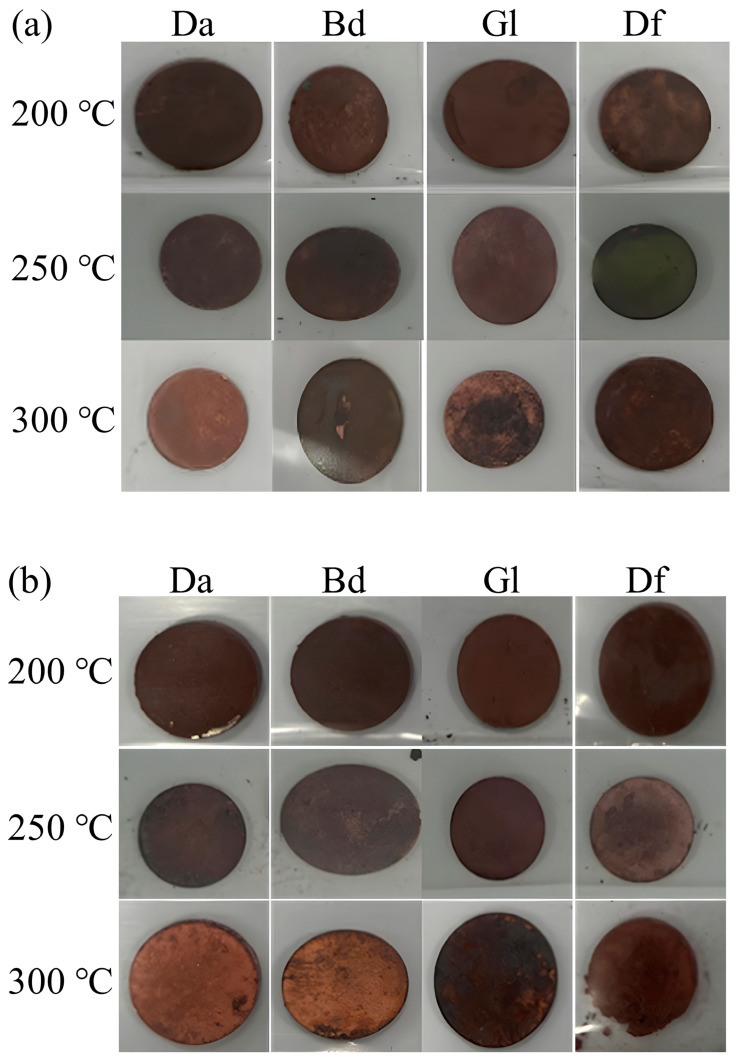
Images of sintered Cu chips: (**a**) MEK and (**b**) MeOH.

**Figure 16:**
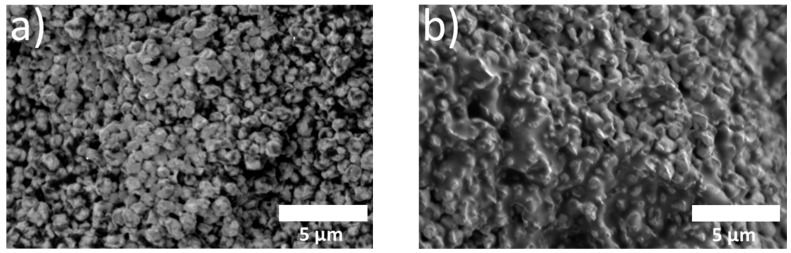
SEM images of fractured surfaces of sintered Cu chips: (**a**) Cu/BA/MEK and (**b**) Cu/BA/Da/MEK.

The fused connectivity of Cu particles within sintered Cu chips plays a pivotal role in determining sintering performance. To assess this, both photographs and SEM images were used to evaluate the morphology of the sintered Cu chips. The Cu sintering paste underwent pre-heating at 200 °C, followed by sintering at different temperatures, 200, 250, and 300 °C, to determine the optimal sintering temperature. Figure 15a,b depict the Cu chips successfully sintered using MEK and MeOH solvents, respectively. Macro-level photographic comparisons of the sintered Cu chips proved challenging, prompting a more in-depth analysis via SEM images, as presented in Figure 16. The fractured surfaces of the sintered Cu chips without and with the incorporation of Da (a representative epoxy) are observed in Figure 16a and 16b, respectively. The sintered Cu chips without Da represented surfaces where Cu appeared closely bonded, minimizing voids and promoting neck formation. Similarly, the incorporation of epoxy reduced the voids but resulted in a less defined morphology. This obscurity in image clarity can be attributed to the interactions stemming from organic reactions.

**Figure 17:**
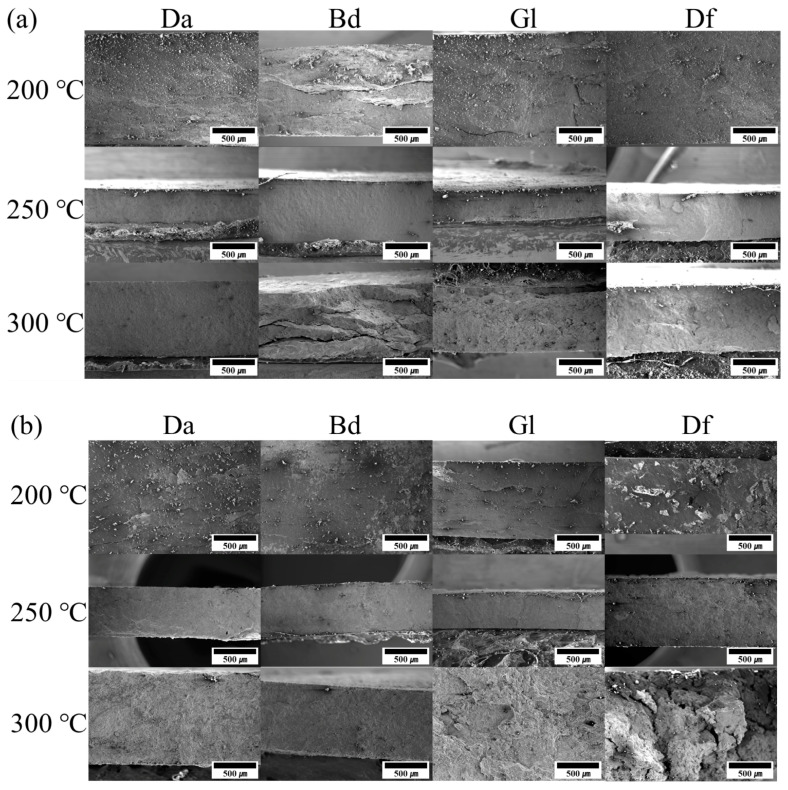
SEM images of fractured surfaces of sintered Cu chips with different epoxy binders, sintering temperatures, and solvents: (**a**) MEK and (**b**) MeOH.

The fractured surfaces of the sintered Cu chips with different epoxy binders, sintering temperatures, and solvents were scrutinized further, as shown in Figure 17 [42,43]. Specifically, Figure 17a,b represent the effects of the MEK and MeOH solvents, respectively. The solvent type had a minimal impact on the morphology. In contrast, discernable variances were observed when different epoxy binders and sintering temperatures were employed. Organic residues were visible for all the samples sintered at 200 °C. In contrast, in the case of the samples sintered at 300 °C, excessive heat under pressure instigated premature epoxy reactions ahead of Cu sintering, resulting in trapped organic residues [44,45]. The sintering temperature of 250 °C led to effective sintering results marked by minimum voids and residues. This efficiency can be attributed to the BA effectively eliminating the Cu oxide layers, the epoxy binders reacting with the BA to yield hydroxyl groups, and the expulsion of most additives and solvents prior to sintering. Consequently, 250 °C was identified as the optimal sintering temperature. The phenomena of residual impurities or the evidence of separation was more pronounced in samples comprising Gl or Bd, as opposed to those with Da and Df. The DSC analysis revealed that the sintering paste containing Gl or Bd triggered reactions at temperatures lower than those infused with Da or Df. This low-temperature reaction caused the epoxy to react prematurely, preceding the Cu sintering. This untimely reaction not only disrupted the sintering process but also led to the persistence of organic residuals in the sintered Cu chip.

### 3.5. Atomic Analysis of Sintered Cu Chips

The removal of Cu oxide and the presence of organic residues within the sintered Cu chips were quantitatively assessed using XPS. An etching procedure was employed to probe the internal composition of these sintered Cu chips. The inner composition, particularly that of the Cu and organic compounds, differed according to the sintering temperature, solvent, and epoxy type. Table 3 and Table 4 summarize the composition distributions of Cu, C, and O in the Cu chips sintered using MEK and MeOH, respectively. In comparison, the Cu chips sintered with MEK showed marginally fewer organic residues than those with MeOH, probably because of the higher boiling point (80 °C) and less polarity of MEK. A key requirement for the sintering process is the uniform dispersion of the acidic additive throughout the mixture to effectively counteract and inhibit the formation of Cu oxide layers before and during the connection of Cu particles. This necessitates a delicate balance involving solvent evaporation timing, polarity, and the associated physical interactions, making the process intricate. In addition, the hydroxyl moiety of MeOH can react with epoxide groups at elevated temperatures, resulting in an increase in organic residues. The mean atomic% of oxygen in the Cu chips sintered using Da and Df was found to be low, which was indirectly confirmed by the SEM morphology observations. The epoxy–acid reactions for Da and Df were initiated at higher temperatures than those for Bd and Gl, thereby allowing for a more seamless merging and sintering of Cu particles. The most efficient sintering temperature was identified to be 250 °C. This observation contradicted the conventional belief that higher sintering temperatures yielded better results. However, based on the results of Table 3 and Table 4, a substantially high sintering temperature caused more oxidation of the Cu, as proven in the TGA results. In contrast, Cu particles did not fuse effectively at lower temperatures. Thus, intermediate sintering temperatures are needed to remove the pre-existing oxidized Cu layers and to prevent new Cu oxide layers caused by heat. For a further analysis of the sintered Cu chips, the sintering temperature of 250 °C was chosen for subsequent analyses in this study.

**Figure 18:**
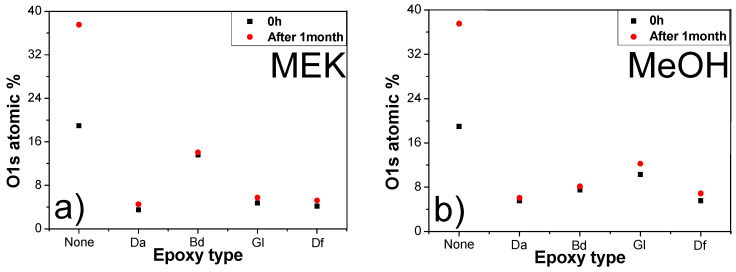
O atomic% of Cu chips sintered using different epoxy and solvent types 0 and 1 month after sintering at 250 °C: (**a**) MEK and (**b**) MeOH.

Oxidation is a concern for sintered Cu chips during their operation. The reacted epoxy has the potential to shield the Cu from oxidation. Figure 18 shows the percentage of oxygen atoms (O atomic%) in the sintered Cu chips with different epoxy types, both immediately (0 day) and 30 days after sintering. Regardless of the solvent type, all the Cu chips sintered using each epoxy additive exhibited an excellent preservation of the Cu against oxidation, compared with the sample devoid of any epoxy. The O atomic% remained largely unchanged after storage for one month. For instance, regarding the oxygen index of the samples prepared with MEK, that without epoxy exhibited an oxygen index of 19.0% immediately after fabrication, which increased to 37.6% after a month. In contrast, the samples with added Da displayed an oxygen index of 3.5% initially, rising marginally to 4.5% after one month. In particular, the sintered Cu chips containing Da or Df exhibited the lowest O atomic% values. This underscores the efficacy of epoxy in curbing the onset of oxide films on Cu sintered chips during their utilization.

**Figure 19:**
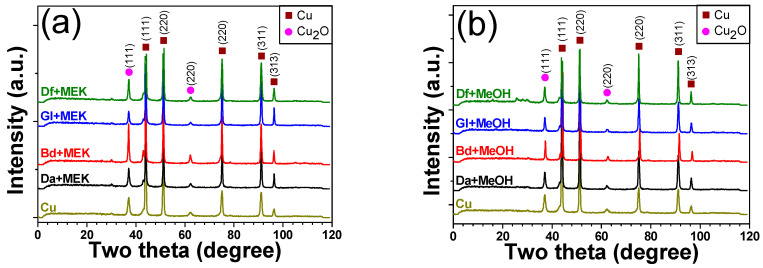
XRD spectra of sintered (at 250 °C) Cu chips with different epoxy and solvent types: (**a**) MEK and (**b**) MeOH.

An XRD analysis was employed as a supplementary technique to corroborate the findings obtained from XPS, as shown in Figure 19. This analysis focused on discerning the presence of Cu oxide layers by identifying peaks corresponding to Cu and Cu_2_O. Peaks appearing at the 2θ values of 43° (111), 51° (220), 75° (220), 91° (311), and 96° (313) were attributed to pure Cu components [46]. Meanwhile, the presence of a peak at 2θ = 37° (111), 62° (220) was indicative of Cu_2_O [46]. Notably, no detectable peaks corresponding to CuO (2θ = 35° and 39° are ascribed to (111) and (022), respectively) were observed in the sintered Cu chips [47]. Typically, Cu_2_O formation occurs at around 250 °C, while CuO tends to form at temperatures ranging from 350 to 450 °C [48]. Among the examined samples, the sintered Cu chip with Da exhibited the most effective removal of Cu oxide, and this observation was consistent across the different solvent types.

### 3.6. Mechanical Properties of Sintered Cu Chips

**Figure 20:**
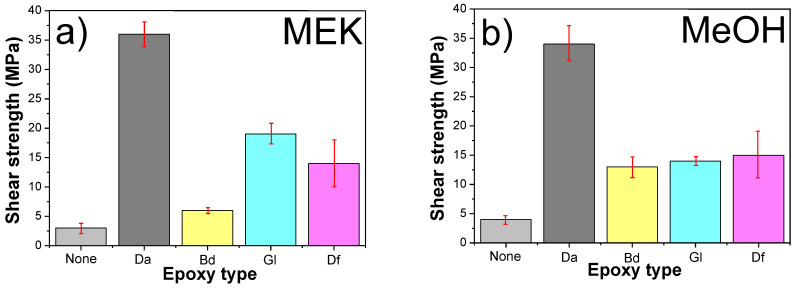
Lab shear strength of Cu chips sintered (250 °C) using different epoxy and solvent types: (**a**) MEK and (**b**) MeOH.

The bonding strength of sintered Cu chips is a crucial parameter for sintering performances. The lab shear strengths of sintered Cu chips with different epoxy and solvent types were evaluated and compared with the sample without epoxy. Figure 20 presents the lab shear strengths of the sintered Cu chips under different conditions. Notably, all epoxy-containing samples exhibited a higher lab shear strength, in comparison to the sample without epoxy, irrespective of the solvent type. For instance, the lab shear strengths of the Cu chips sintered using MEK were as follows: 2.7 MPa (no epoxy), 35.9 MPa (Da), 5.4 MPa (Bd), 13.4 MPa (Gl), and 15.3 MPa (Df). Remarkably, the highest strength was achieved for the Da-containing sample. The Cu particles were effectively sintered together prior to the epoxy reactions between the Da and acidic moieties. Subsequent to sintering, a durable 3D structure, facilitated by the epoxy reactions between the Da and acidic groups, emerged on the sintered Cu chip surfaces, thereby enhancing the strength. The trend in lab shear strengths for samples using MeOH mirrored those formulated with MEK. Table 5 and Table 6 show a summary of the electrical and thermal conductivities, and the O atom% of the Cu sintered chips with different epoxy binders immediately and 1 month after sintering by using MEK and MeOH, respectively.

## 4. Conclusions

The increasing demand for enhanced electronic devices underscores the essential role of semiconductor packaging in semiconductor manufacturing and design. Semiconductor packaging safeguards the fragile semiconductor device and ensures necessary electrical connections between the semiconductor device and external circuitry. This study investigated the interaction between epoxy (Da, Bd, Gl, and Df) and acidic additives in the sintering process of semiconductor devices. Six methods of achieving electrical interconnections were highlighted, with sintering being notably effective for specific applications, especially those demanding high thermal conductivity and stability at elevated temperatures. The sintering process, particularly with Cu due to its cost-effectiveness and superior conductivity properties, demands meticulous conditions to achieve optimal results. One notable challenge with Cu is the formation of oxide layers, which need to be removed before bonding. Epoxy, commonly used for its robust mechanical properties and adhesion capabilities, was explored as an effective element to enhance the sintering performances, in particular the bonding strength. This study revealed not only the combination of ingredients but also the sintering conditions. We achieved the following findings: The mixing method influenced the stability of the Cu paste. Phase separation was observed in the Cu sintering pastes with varying solvent concentrations, underlining the importance of the acidic additive concentration. A thermal analysis determined the optimal pre-heating (200 °C) and sintering (250 °C) temperatures. The electrical conductivity of the sintered Cu chips was influenced by the epoxy ratio, with increased the epoxy content, leading to reduced conductivity. Morphological studies via SEM revealed the importance of the sintering temperature in achieving optimal results. An atomic analysis provided insights into the effectiveness of epoxy in preventing the oxidation of Cu in sintered chips. In the samples devoid of epoxy, the oxygen index exhibited a reduction exceeding fifty percent. Conversely, in the samples including the epoxy, the oxygen index remained largely stable. The incorporation of epoxy into the Cu sintered chips enhanced various properties including the electrical, thermal, and mechanical properties for long-term applications, owing to the oxidative inhibition of the epoxy. The mechanical properties, especially the bonding strength, were enhanced to a peak value of 35.9 MPa when epoxy was integrated into the sintering process. The sintered samples with bisphenol-based epoxy binders exhibited the lowest oxygen concentration and the highest electrical/thermal conductivities after 1 month. In summary, the addition of a small amount of epoxy (20 wt%) in the sintering process of semiconductor devices is beneficial, as it improves mechanical robustness while maintaining electrical conductivity with minimal reduction. The findings from this study have the potential to influence future approaches in semiconductor packaging, emphasizing the balance between the composition and processing conditions for achieving high-performance outcomes. In particular, this Cu sintering technology with excellent electrical and thermal conductivities and with reliability can be used in various applications requiring finer pitches and smaller geometries, such as die attach materials, high-power devices, light emitting diode (LED) packaging, automotive electronics, 5G technology, integrated circuits (ICs), microelectromechanical systems (MEMSs), and radio frequency (RF) and microwave devices.

## Figures and Tables

**Table 1:** Contents of each component with different epoxy/acid ratios.

Epoxy: Acid	Epoxy (g)	Acid (g)	Solvent (mL)	Cu Powder (g)
0:100	0	1	2.3	4
20:80	0.2	0.8	1.8	4
40:60	0.4	0.6	1.3	4
60:40	0.6	0.4	0.8	4
80:20	0.8	0.2	0.3	4

**Table 2:** Correction factor of circularshaped sample for pin spacing; C(D/S) [35]. Reprinted/adapted with permission from Ref. [35] [Smits, F. M. Measurement of Sheet Resistivities with the Four-Point Probe. Bell Syst. Tech. J. 1958, 37 (3), 711–718]. Copyright 1958 Nokia Corporation and AT&T Archives.

D/S	Correction Factor of Circular-Shaped Sample Size for Pin Spacing; C
4.0	2.9289
5.0	3.3625
7.5	3.9273
10.0	4.1716
15.0	4.3646
20.0	4.4364
40.0	4.5076
∞	4.5324

**Table 3:** Atomic% of the inside of the Cu chips sintered using MEK with different epoxy binders and sintering temperatures ^a^.

Name	Without Epoxy	Da			Bd			Gl			Df		
°C ^a^	250	200	250	300	200	250	300	200	250	300	200	250	300
Cu2p	81.1	14.8	65.4	47.7	14.4	76.6	57.6	12.2	70.9	63.7	39.9	83.8	53.6
C1s	-	79.3	31.1	44.9	80.6	9.8	31.2	82.2	24.3	31.5	56.0	12.1	42.3
O1s	19.0	5.85	3.48	7.33	5.01	13.6	11.2	5.6	4.8	4.9	4.4	4.2	4.1

**Table 4:** Atomic% of the inside of the Cu chips sintered using MeOH with different epoxy binders and sintering temperatures ^a^.

Name	Without Epoxy	Da			Bd			Gl			Df		
°C ^a^	250	200	250	300	200	250	300	200	250	300	200	250	300
Cu2p	81.1	31.4	33.1	57.0	15.8	46.6	4.6	12.2	37.6	37.8	39.6	42.7	74.4
C1s	-	63.7	61.4	34.1	78.9	45.9	84.1	82.2	52.1	17.3	56.0	51.8	5.4
O1s	19.0	4.9	5.6	8.9	5.4	7.5	11.3	5.6	10.3	45.0	4.4	5.6	20.2

**Table 5:** Electrical and thermal conductivities and O atomic% of Cu sintered chips with different epoxy binders using MEK immediately and 1 month after sintering.

	Electrical Conductivity (S/m)		Thermal Conductivity (W/m·K)		O Atomic % (%)	
	0 h	1 month	0 h	1 month	0 h	1 month
None	2.00 × 10³	0.80 × 10³	249	111	19.0	37.6
Da	1.20 × 10³	1.00 × 10³	188	161	3.5	4.5
Bd	1.22 × 10³	0.84 × 10³	147	109	13.6	14.1
Gl	1.24 × 10³	0.88 × 10³	145	107	4.8	5.7
Df	1.24 × 10³	1.04 × 10³	181	155	4.2	5.2

**Table 6:** Electrical and thermal conductivities and O atomic% of Cu sintered chips with different epoxy binders using MeOH immediately and 1 month after sintering.

	Electrical Conductivity (S/m)		Thermal Conductivity (W/m·K)		O Atomic % (%)	
	0 h	1 month	0 h	1 month	0 h	1 month
None	2.50 × 10³	0.90 × 10³	241	110	19.0	37.6
Da	2.00 × 10³	1.72 × 10³	175	148	5.6	6.1
Bd	1.20 × 10³	0.90 × 10³	151	111	7.5	8.2
Gl	1.00 × 10³	0.90 × 10³	145	101	10.3	12.3
Df	1.20 × 10³	1.11 × 10³	168	138	5.6	6.9

## Data Availability

Data are contained within the article.
